# Supplementary material for: Mental health related stigma in a primary care setting in Karnataka, rural India: Service user and carer perspectives
Source: PLoS One. 2025 Aug 22;20(8):e0330949. doi: 10.1371/journal.pone.0330949 (PMC12373246; doi:10.1371/journal.pone.0330949)
Supplement: S1 File — (PDF) [file pone.0330949.s001.pdf]

INDIGO-PRIMARY: PRogramme for IMplementing interventions addressing knowledge, Attitudes and behaviour of primaRY care providers towards people with mental illness

---

Topic guide 5

Interviews with service users (SU) and their family members

(Several of the questions below have been used previously in a study of service users' and family caregivers' experiences of stigma and discrimination in India (Koschorke et al., 2014))

Interviews with service users

General questions

- Can you tell me a little about yourself? Probes for demographics (age, socioeconomic background, ethnicity, etc.)
- Where do you live? Probe: how far to access PHC and specialist services, transport, any obstacles for attending the PHC for care?

Type of mental health problems, explanatory models, help-seeking and possible reasons for delays in helpseeking

- Can you tell me about the problems you are receiving treatment for at this PHC?
  - o When did you first notice these problems?
  - o What did you do when this started? /What did your family do? [establish approximate process of helpseeking]
  - o How long have you been receiving treatment for at this PHC?
  - o If delayed helpseeking; probe for possible reasons including not knowing treatment available/ sought help first from other sources of healing/ fears of stigma and discrimination
- What do you call these problems you are getting treatment for?
- What do you think has caused these problems?

Experiences with treatment

- What treatments are you receiving at the moment? From whom? Have you received treatment from the PHC only or also other sources? (probe: mental health services and hospitals, other healers)
- What has your experience been with the treatment you have been getting so far? *[probing for treatments received so far that were mentioned earlier, including PHC and specialist treatments]*
  - What did you like about it? What did you not like?
  - How do you feel about the treatment you are getting at the moment?
  - How is it for you when you go and see your doctor/nurse/health worker?

[Probe for: interaction with doctor/health worker; feeling respected; continuity or lack of continuity of care; whether or not enough time available to speak about mental health problems, worries about being seen by the doctor/health worker, worries about other patients finding out about the condition through visits at the PHC, etc

Experiences of stigma and discrimination

- Do you know anyone else who is having similar problems to yours?
  - Can you tell me about it?

- What do you think other people say/think about people who have this illness?
    - How do they react? How do they treat them?
    - Have you experienced anything like that? (If yes: Can you give me examples? How did this affect you?)
- Suppose there is one person who has a similar problem to yours and another person who has diabetes:
    - Will people treat him/her the same or different? How about healthcare staff, will they treat him/her the same or different?

If different:

- Why? What is it about the illness that is different?
- Have you experienced anything like this?

- Some people tell me that people with health problems like yours are sometimes treated differently – what do you think? (where appropriate, use terms stigma and discrimination)
    - Has anything like that happened to you? Can you give me examples?
- Has anything like that happened to you
    - when you were attending the PHC centre?
    - when you were attending specialist services for mental health?
    - Probe for specific experiences, e.g. treated with less respect, being shouted at, not listened to, not taken serious, not being given the treatments required, restraints, verbal and physical abuse, etc.
    - When this happened, how did you feel? What impact did it have on you?
    - When this happened what did you do? Did you receive any help or support? From where?

Resources and anti-stigma interventions

- If it happened to you in the future that you were treated differently or had a complaint, what resources do you think are available to address this?
- Are there advocacy/human rights/ or support organizations that support people like yourself and your families? If, so what support or training have you received from them?
- Have you ever been involved in providing training yourself? Where was that? To whom?
- Have you been involved in any interventions to reduce stigma and discrimination? What were your experiences?
- When PHC staff are trained to treat people with mental illness in the best possible way, what do you think would be important for them to be taught about? Do you have any other suggestions for what would make a positive difference for people with problems like yours in primary care?

Interviews with family members

The questions outlined above for service users will also be addressed at family members, i.e., family members will be asked about their perceptions of service users experiences of treatment and experiences of stigma and discrimination.
