# Supplementary material for: Mental health related stigma in a primary care setting in Karnataka, rural India: Service user and carer perspectives
Source: PLoS One. 2025 Aug 22;20(8):e0330949. doi: 10.1371/journal.pone.0330949 (PMC12373246; doi:10.1371/journal.pone.0330949)
Supplement: S2 File — (PDF) [file pone.0330949.s002.pdf]

Inclusivity in global research

PLOS' policy on inclusivity in global research aims to improve transparency in the reporting of research performed outside of researchers' own country or community and ensures that PLOS publications reporting global research adhere to high standards for research ethics and authorship. Authors of relevant research articles may be asked to complete the questionnaire below, which outlines ethical, cultural, and scientific considerations specific to inclusivity in global research. This questionnaire may be requested when researchers have travelled to a different country to conduct research, if research uses samples collected in another country, research with Indigenous populations or their lands, or if research is on cultural artefacts. Researchers travelling to another country solely to use laboratory equipment will not normally be required to complete the questionnaire. However, the questionnaire can be requested at the journal's discretion for any submission – if you have been requested to complete this questionnaire by the PLOS journal you submitted to, please do so.

Please complete the questionnaire below and include this as a Supporting Information file with your manuscript. Note that if your paper is accepted for publication, this checklist will be published with your article in the supporting information files. Please ensure that you reference the checklist in the main body of your manuscript. We suggest adding a subsection 'Inclusivity in global research' to your Methods section and adding the following sentence: "Additional information regarding the ethical, cultural, and scientific considerations specific to inclusivity in global research is included in the Supporting Information (SX Checklist)"

The questions have been designed to be applicable to a wide range of study types, and there are subsections for both human subjects research and non-human subjects research. If any of the questions are not relevant to your research please mark them as "N/A" as appropriate.

Ethical considerations, permits and authorship

This section is applicable to all research types.

Provide details as to who granted permissions and/or consent for the study to take place in the Methods section of your manuscript. This should include the names of **all** ethics boards, governmental organizations, community leaders or other bodies that provided approval for the study. If individuals provided approval refer

to these people by their role or title but do not list their name(s).

Reported on page number: 7 in manuscript with track changes.

Ethics approval was obtained from the PNM Research Ethics Subcommittee, King's College, London: Reference Number (No.) RESCMR-17/18-4109; approval date 23 February 2017 and NIMHANS Institutional Review Board: Reference Number (No.) NIMHANS/IEC(BEH.SC.DIV.)7th MEETING/2017. Permission was granted from Directorate of Health Family Welfare Servicers, Government of Karnataka, India, No: DD/Mental Health/10/18-19 dated 26.04.2018.

If there were any deviations from the study protocol after approval was obtained please provide details of these changes in the Methods section of your manuscript.

Reported on page number:

N/A; there were no deviations from th study protocol.

Did this study involve local collaborators that are residents of the country where the research was conducted or members of the community studied? If you do not have any authors from said communities, please provide an explanation for this below.

Everyone listed as an author should meet PLOS' criteria for authorship and all individuals who meet these criteria should be included in the author byline, rather than the acknowledgements. For further information please see the journal's Authorship Policy.

Yes, local collaborators from Karnataka, India, were centrally involved as collaborators in this research, and likewise included as authors (including as joint senior author), as per author details. We confirm that everyone listed as an author meets the criterial for authorship.

Human subjects research (e.g. health research, medical research, cross-cultural psychology)

Did you obtain written informed consent from a representative of the local community or region before the research took place? How did you establish who speaks for the community? Details of written informed consent obtained from study participants should be reported separately in the Methods section of your manuscript.

Yes, approval for conducting this research was provided by NIMHANS Institutional Review Board: Reference Number (No.) NIMHANS/IEC(BEH.SC.DIV.)7th MEETING/2017. Permission was granted from Directorate of Health Family Welfare Servicers, Government of Karnataka, India, No: DD/Mental Health/10/18-19 dated 26.04.2018. These details are provided in the 'Ethical approval' section of the methods.

The 'Participants and procedure' section in the methods specifies that "Participation in the study was initiated only after written, informed consent had been obtained.".

How did members of the local community provide input on the aims of the research investigation, its methodology, and its anticipated outcome(s)?

The project team included colleagues from and working in the local community, and the project was designed in collaboration with these colleagues, including shaping project methodology and considering anticipated outcomes. As this manuscript reports on site-specific data colleged within a larger multi-site project, local collaborators from all research sites were involved in shaping the broader project, i.e. the project was not designed to address issues specific to Karnataka, India, only, but rather broader cross-cutting issues present across a range of research sites.

When engaging with the local community, how did you ensure that the informed consent documents and other materials could be understood by local stakeholders?

The ethics materials – including information sheets and consent documents - were reviewed and adjusted by the local research team in view of acceptability and comprehensibility for the local study population in Karnataka, India. As described for a previous question, local approval procedures reviewed and approved the use of these materials. These approval procedures involved establishing that the materials could be understood by local stakeholders.

Will the findings of the research be made available in an understandable format to stakeholders in the community where the study was conducted (e.g. via a presentation, summary report, copies of publications, etc.)? Please provide details of how this will be achieved.

We are pleased to share that we have been actively engaged in the same community and working with the same population for multiple years since the data for this study were collected.

The findings from our the research reported in this manuscript have played a significant role in shaping the cultural adaptation of interventions implemented as a part of the Indigo Partnership research programme which was conducted in the same locality (see Gronholm et al., 2023; Toward a multi-level strategy to reduce stigma in global mental health: overview protocol of the Indigo Partnership to develop and test interventions in low- and middle-income countries). These interventions primarily benefited ASHA workers and service providers from primary health care settings.

Furthermore, the outcomes of the study have been incorporated into anti-stigma training sessions conducted with staff from various primary health care centers.

Looking ahead, we plan to provide brief summary reports to each primary health care center that participated in the study, ensuring continued collaboration and knowledge sharing.

Non-human subjects research using specimens/ animals collected as part of the study, or those housed in archival collections. Examples include archaeology, paleontology, botany and zoology.

Did the permission you obtained from a local authority to perform the study include an agreement on access to outputs and benefit sharing? This may include procedures to enable fair distribution of the benefits and resources arising from the research performed. Please include any details of Prior Informed Consent and Benefit Sharing Agreements obtained. These may be required by field-specific regulations, for example the Convention on Biological Diversity (CBD) and the associated Nagoya Protocol.

n/a

If the material used in your study was imported, please A) provide the year it was imported and B) indicate whether permits were obtained to import/export the materials used, C) provide details of any permits obtained. If this information is not available, please indicate this.

n/a

If you used archival specimens, please state how the material used in your study was acquired by the institute it is held in and provide details of any permits obtained for the original excavations/ sample collection. If this information is not available, please indicate this.

n/a

How was the potential cultural significance of the materials collected in your study to local communities considered in your research design? Were Indigenous peoples and/or local researchers and institutions involved with archaeological excavations / collection of specimens? If so, please provide a description of their involvement.

n/a

If your manuscript includes photographs of human remains please indicate whether authors obtained permission from descendants or affiliated cultural communities to do so.

n/a
