# Supplementary material for: Mental health related stigma in a primary care setting in Karnataka, rural India: Service user and carer perspectives
Source: PLoS One. 2025 Aug 22;20(8):e0330949. doi: 10.1371/journal.pone.0330949 (PMC12373246; doi:10.1371/journal.pone.0330949)

PLOS ONE

Mental health related stigma in a primary care setting in rur[illegible]ndia: service user and carer perspectives

--Manuscript Draft--

**Manuscript Number:**	PONE-D-24-43039
**Article Type:**	Research Article
**Full Title:**	Mental health related stigma in a primary care setting in rural India: service user and carer perspectives
**Short Title:**	Mental health related stigma in a primary care setting in rural India: service user and carer perspectives
**Corresponding Author:**	Petra C Gronholm<br>King's College London Institute of Psychiatry Psychology & Neuroscience<br>London, UNITED KINGDOM OF GREAT BRITAIN AND NORTHERN IRELAND
**Keywords:**	mental health; stigma; low-middle income countries; India; service user perspective
**Abstract:**	Mental health related stigma and discrimination are significant issues requiring urgent global action. There is a particular lack of research on stigma in low-and middle-income countries. This study explores the experiences of service users and their carers who acces[illegible]imary care for treatment of mental health conditions in rural Karnataka, India. Purposive sampling was used to conduct qualitative interviews of service users and their carers. This study highlights several barriers to access mental healthcare that individuals face on a system level. The results also bring to light pervasive experiences of stigmatising attitudes and discriminatory behaviour that participants with mental health conditions face from within their communities. Cohesion within both the family and the community was highlighted as a priority for participants, using a “what matters most” approach. This study brings insights to contribute to the development of locally relevant anti-stigma interventions, promoting access to care, and the human rights and quality of life for individuals with mental health conditions in a rural community in Karnataka, India.
**Order of Authors:**	Sarah J Parry
	Gurucharan Bhaskar Mendon
	Mirja Koschorke
	Adarsha Alur Manjappa
	Arya Thirumeni
	Graham Thornicroft
	Anish V Cherian
	Petra C Gronholm
**Additional Information:**	
**Question**	**Response**
**Financial Disclosure**<br><br>Enter a financial disclosure statement that describes the sources of funding for the work included in this submission. Review the submission guidelines for detailed requirements. View published research articles from *PLOS ONE* for specific examples.	Yes

Powered by Editorial Manager® and ProduXion Manager® from Aries Systems Corporation

This statement is required for submission and **will appear in the published article** if the submission is accepted. Please make sure it is accurate.<br><br>**Funded studies**<br>Enter a statement with the following details:<br>• Initials of the authors who received each award<br>• Grant numbers awarded to each author<br>• The full name of each funder<br>• URL of each funder website<br>• Did the sponsors or funders play any role in the study design, data collection and analysis, decision to publish, or preparation of the manuscript?<br><br>Did you receive funding for this work?	
Please add funding details.<br>as follow-up to "**Financial Disclosure**<br><br>Enter a financial disclosure statement that describes the sources of funding for the work included in this submission. Review the submission guidelines for detailed requirements. View published research articles from *PLOS ONE* for specific examples.<br><br>This statement is required for submission and **will appear in the published article** if the submission is accepted. Please make sure it is accurate.<br><br>**Funded studies**<br>Enter a statement with the following details:<br>• Initials of the authors who received each award<br>• Grant numbers awarded to each author<br>• The full name of each funder<br>• URL of each funder website<br>• Did the sponsors or funders play any role in the study design, data collection and analysis, decision to publish, or preparation of the manuscript?	GT, MK, and PCG were supported by the UK Medical Research Council (UKRI) for the Indigo Partnership (MR/R023697/1) award. GT has recently been supported by the National Institute for Health and Care Research (NIHR) Applied Research Collaboration South London (NIHR ARC South London) at King's College Hospital NHS Foundation Trust. The views expressed are those of the author(s) and not necessarily those of the NIHR or the Department of Health and Social Care. For the purpose of open access, the author has applied a Creative Commons Attribution (CC BY) licence (where permitted by UKRI, 'Open Government Licence' or 'Creative Commons Attribution No-derivatives (CC BY-ND) licence' may be stated instead) to any Author Accepted Author Manuscript version arising from this submission.<br><br>AC received funding from an Intra mural grant from National Institute of Mental Health and Neuroscience, Bengalaru, India.

Did you receive funding for this work?"	
Please select the country of your main research funder (please select carefully as in some cases this is used in fee calculation).<br>as follow-up to "**Financial Disclosure**<br><br>Enter a financial disclosure statement that describes the sources of funding for the work included in this submission. Review the submission guidelines for detailed requirements. View published research articles from *PLOS ONE* for specific examples.<br><br>This statement is required for submission and **will appear in the published article** if the submission is accepted. Please make sure it is accurate.<br><br>**Funded studies**<br>Enter a statement with the following details:<br>• Initials of the authors who received each award<br>• Grant numbers awarded to each author<br>• The full name of each funder<br>• URL of each funder website<br>• Did the sponsors or funders play any role in the study design, data collection and analysis, decision to publish, or preparation of the manuscript?<br><br>Did you receive funding for this work?"	UNITED KINGDOM - GB
**Competing Interests**<br><br>Use the instructions below to enter a competing interest statement for this submission. On behalf of all authors, disclose any competing interests that could be perceived to bias this work—acknowledging all financial support and any other relevant financial or non-financial competing interests.<br><br>This statement is required for submission and **will appear in the published article** if	The authors have declared that no competing interests exist.

the submission is accepted. Please make sure it is accurate and that any funding sources listed in your Funding Information later in the submission form are also declared in your Financial Disclosure statement.<br><br>View published research articles from *PLOS ONE* for specific examples.<br><br>**NO authors have competing interests**<br><br>Enter: *The authors have declared that no competing interests exist.*<br><br>**Authors with competing interests**<br><br>Enter competing interest details beginning with this statement:<br><br>*I have read the journal's policy and the authors of this manuscript have the following competing interests: [insert competing interests here]*<br><br>* typeset	
**Ethics Statement**<br><br>Enter an ethics statement for this submission. This statement is required if the study involved:<br><br>• Human participants<br>• Human specimens or tissue<br>• Vertebrate animals or cephalopods<br>• Vertebrate embryos or tissues<br>• Field research<br><br>Write "N/A" if the submission does not require an ethics statement.<br><br>General guidance is provided below. Consult the submission guidelines for detailed instructions. **Make sure that all information entered here is included in the Methods section of the manuscript.**	Ethical approval was obtained from the PNM Research Ethics Subcommittee, King's College, London: Reference Number (No.) RESCMR-17/18-4109 and NIMHANS Institutional Review Board: Reference Number (No.) NIMHANS/IEC(BEH.SC.DIV.)7th MEETING/2017. No further ethical clearance was required, as permission for the use of these data in further studies including secondary analyses was already obtained through the original ethical approval.

**Format for specific study types**<br><br>**Human Subject Research (involving human participants and/or tissue)**<br>• Give the name of the institutional review board or ethics committee that approved the study<br>• Include the approval number and/or a statement indicating approval of this research<br>• Indicate the form of consent obtained (written/oral) or the reason that consent was not obtained (e.g. the data were analyzed anonymously)<br><br>**Animal Research (involving vertebrate animals, embryos or tissues)**<br>• Provide the name of the Institutional Animal Care and Use Committee (IACUC) or other relevant ethics board that reviewed the study protocol, and indicate whether they approved this research or granted a formal waiver of ethical approval<br>• Include an approval number if one was obtained<br>• If the study involved *non-human primates*, add *additional details* about animal welfare and steps taken to ameliorate suffering<br>• If anesthesia, euthanasia, or any kind of animal sacrifice is part of the study, include briefly which substances and/or methods were applied<br><br>**Field Research**<br><br>Include the following details if this study involves the collection of plant, animal, or other materials from a natural setting:<br>• Field permit number<br>• Name of the institution or relevant body that granted permission	
**Data Availability**<br><br>Authors are required to make all data underlying the findings described fully available, without restriction, and from the time of publication. PLOS allows rare exceptions to address legal and ethical concerns. See the PLOS Data Policy and FAQ for detailed information.	No - some restrictions will apply

A Data Availability Statement describing where the data can be found is required at submission. Your answers to this question constitute the Data Availability Statement and **will be published in the article**, if accepted.<br><br>**Important:** Stating 'data available on request from the author' is not sufficient. If your data are only available upon request, select 'No' for the first question and explain your exceptional situation in the text box.<br><br>Do the authors confirm that all data underlying the findings described in their manuscript are fully available without restriction?	
**Describe where the data may be found in full sentences. If you are copying our sample text, replace any instances of XXX with the appropriate details.**<br><br>• If the data are **held or will be held in a public repository**, include URLs, accession numbers or DOIs. If this information will only be available after acceptance, indicate this by ticking the box below. For example: *All XXX files are available from the XXX database (accession number(s) XXX, XXX.*).<br>• If the data are all contained **within the manuscript and/or Supporting Information files**, enter the following: *All relevant data are within the manuscript and its Supporting Information files.*<br>• If neither of these applies but you are able to provide **details of access elsewhere**, with or without limitations, please do so. For example:<br><br>*Data cannot be shared publicly because of [XXX]. Data are available from the XXX Institutional Data Access / Ethics Committee (contact via XXX) for researchers who meet the criteria for access to confidential data.*<br><br>*The data underlying the results presented in the study are available from (include the name of the third party*	Data cannot be shared publicly because informed consent was not sought for sharing the data through other means than via request from the author. Data are available from authors via request. No reasonable request will be denied.

and contact information or URL).<br>• This text is appropriate if the data are owned by a third party and authors do not have permission to share the data.<br><br>* typeset	
Additional data availability information:	

Manuscript

Click here to access/download;Manuscript;Manuscript_Mental health stigma primary care rural India.docx

Mental health related stigma in a primary care setting in rural India: service user and carer perspectives

Sarah J Parry[1], Gurucharan Bhaskar Mendon[2], Mirja Koschorke[3], Adarsha Alur Manjappa[4], Arya Thirumeni[2], Graham Thornicroft[5], Anish V Cherian[2&] & Petra C Gronholm*[5,6&]

[1]South London and Maudsley NHS Foundation Trust, London, United Kingdom

[2]Department of Psychiatric Social Work, National Institute of Mental Health and Neurosciences, Bengaluru, India

[3]Centre for Global Mental Health, Institute of Psychiatry, Psychology & Neuroscience, King's College London, United Kingdom

[4]District Mental health Program (DMHP), Department of Health and Family Welfare, Karnataka, India

[5]Centre for Global Mental Health and Centre for Implementation Science, Health Service and Population Research Department, Institute of Psychiatry, Psychology & Neuroscience, King's College London, United Kingdom

[5]Centre for Global Mental Health, Department of Population Health, London School of Hygiene & Tropical Medicine, London, United Kingdom

*Corresponding author: petra.gronholm@kcl.ac.uk

Abstract

Mental health related stigma and discrimination are significant issues requiring urgent global action. There is a particular lack of research on stigma in low-and middle-income countries. This study explores the experiences of service users and their carers who access primary care for treatment of mental health conditions in rural Karnataka, India. Purposive sampling was used to conduct qualitative interviews of service users and their carers. This study highlights several barriers to access mental healthcare that individuals face on a system level. The results also bring to light pervasive experiences of stigmatising attitudes and discriminatory behaviour that participants with mental health conditions face from within their communities. Cohesion within both the family and the community was highlighted as a priority for participants, using a "what matters most" approach. This study brings insights to contribute to the development of locally relevant anti-stigma interventions, promoting access to care, and the human rights and quality of life for individuals with mental health conditions in a rural community in Karnataka, India.

Introduction

Stigma and discrimination experienced by people with mental health conditions are often said to be worse than the condition itself and contribute to barriers in accessing healthcare, employment and education, and damages relationships (1). The way stigma and discrimination are experienced varies within different societies and cultures, according to what matters most in that particular context (2). The experience of stigma negatively impacts

upon recovery outcomes as well as on quality of life for people with mental health conditions and is a barrier to access and provision of care (1, 3).

It is widely recognised that there is a stark treatment gap between the number of people with mental health conditions worldwide and those accessing treatment, particularly in low- and middle-income countries (LMIC)(4). There is a significant discrepancy globally between the priority of physical health care and mental health care, with mental health care often taking a lower priority in terms of budget and policy (1, 5). The stigma associated with seeking care for mental health conditions can inadvertently reinforce this treatment gap by reducing the demand for care, and the lack of demand reduces the pressure to raise the priority of mental health care funding and to support a greater supply of services (1, 5).

Stigma has been shown to be a factor in delayed help-seeking in mental health (6). However, the way stigma affects individuals, communities and healthcare systems varies between societies and is less well understood in LMICs where less research has been conducted (1). Thus, as well as a treatment gap for mental health, there is a significant research gap in LMIC (5), although the evidence base in LMICs is emerging (7, 8). The recent Lancet Commission on Ending Stigma and Discrimination in Mental Health highlighted the importance of research focused on the perspectives of service users and carers, especially in LMICs (1). Exploring the lived experience of people with mental health conditions in LMICs is crucial to understand the complexities underlying the gap between the number of people requiring and accessing support for their mental health. High quality qualitative research sheds light on the nature of the barriers and facilitators related to the accessibility, quality and effectiveness of mental healthcare.

India's population was estimated to be approximately 1.4 billion in 2023, equivalent to nearly a fifth of the total world population (9). India is governed by a federal system and comprises

of 28 states and 8 union territories with healthcare being governed by the union and state governments (10). India has private and public healthcare providers, as well as non-governmental organisations providing healthcare, with the majority of private healthcare located in urban centres and rural areas mainly being served by the public system (11). There is a three-tier system of public community care with the most local service being primary health centres (PHC) serving populations of 20-30,000, community health centres (CHC) serving 80-120,000 and tertiary health care (10). Primary health centres may follow up individuals who have received diagnoses at a tertiary level by specialists and have long-term close therapeutic relationships with the communities they serve.

In 2017, nearly 200 million people in India were estimated to have a mental disorder – 1 in every 7 people (12). From 1990-2017, the proportion of people with mental disorders contributing to overall disease burden in India doubled. Access to care varies from state to state and India's first national mental health policy was launched in 2014 prior to the 2017 revised Mental Health Act (13). The treatment gap for common mental disorders has been estimated to be approximately 80% (14). India has approximately 0.75 psychiatrists per 100,000 people (15). Mental health conditions are mainly diagnosed at a tertiary or district level by psychiatrists as well as via the District Mental Health Programme. Medical officers at primary health centres are trained to identify mental health conditions and provide follow up with service users including ongoing prescribed treatment.

Given this context, the aim of this study was to explore the experiences of stigma and discrimination of service users, and their carers, attending a primary health centre in rural Karnataka, India for mental health conditions. The results of this study contribute to understanding the barriers in accessing mental health services that individuals face in rural communities such as Karnataka in India. Such insights can inform the development of stigma

reduction interventions and contribute to the enhancement of the rights and quality of life of individuals facing mental health conditions, and improve access to care in the future.

Methods

Study design

This study used data collected as part of the Indigo PRIMARY study (16), a multisite qualitative study of experiences of stigma relating to mental health conditions in primary care settings across Asia, Africa and Europe. These data have previously been analysed considering data from all research sites (16). The purpose of this study is to carry out a focused and more detailed analysis of the data collected with service-users and carers in the site in India specifically. The site for this study was in rural India, in Ramanagara district within Karnataka state, at PHC[illegible]sociated with the District Mental Health Programme.

Participants and procedure

Participants were recruited through purposive sampling by staff at PHCs who identified people receiving care for their mental health at the PHC and their carers. Those who were willing to participate were included and service users who were currently symptomatic or cognitively impaired were excluded. Participation in the study was initiated only after providing written, informed consent was provided. The sample size was dictated by the data collected in the Indigo PRIMARY study, where a sample of around 10-15 participants per research site was assessed as sufficient to reach data saturation (17).

The recruitment and data collection took place between 1st June 2018 and 31st July 2019. Interviews were carried out by members of the research team from National Institute for Mental Health and Neurosciences (NIMHANS) in the Kannada language.

Interviews lasted 60-90 minutes and a semi-structured approach was used, with an interview guide addressing topics related to experiences of stigma within healthcare and community settings for service users and carers. Questions used in the interviews are available as supplementary material. The interviews were translated and then transcribed in English by the same researcher. A further researcher, fluent in both English and Kannada crosschecked the transcript with audio recordings and reviewed the translation independently.

Data analysis

The transcribed data were inputted to NVivo (R14.23.2) and examined through thematic analysis. An inductive method of thematic analysis was used to generate codes, identify themes and refine them to final key themes (18). Analysis was carried out by author SP in collaboration with the three other authors (AC, PCG, GM), to identify themes describing the entire data set. All key themes were developed and agreed on by all four authors over a series of online meetings.

Ethical approval

Ethical approval was obtained from the PNM Research Ethics Subcommittee, King's College, London: Reference Number (No.) RESCMR-17/18-4109 and NIMHANS Institutional Review Board: Reference Number (No.) NIMHANS/IEC(BEH.SC.DIV.)7th MEETING/2017. No further ethical clearance was required, as permission for the use of these data in further studies including secondary analyses was already obtained through the original ethical approval.

Results

A total of 14 qualitative interviews were carried out with nine service users and five carers. Demographic data was collected for the participants, detailed in Table 1 and 2

Table 1: Demographic details of service users (n=9)

**Gender**	
Male	3
Female	6
**Age (years)***	
<30	0
31-40	3
41-50	1
51-60	1
61-70	2
>70	1
**Marital Status**	
Married	9
Single	0
**Religion**	
Hindu	9
Other	0

*one participant did not provide details of age

Table 2: Demographic details of carers (n=5)

**Gender**	
Male	3
Female	2
**Age (years)**	
<30	0
31-40	1
41-50	1
51-60	2
61-70	1
>70	0
**Marital Status**	
Married	5
Single	0
**Religion**	
Hindu	5
Other	0
**Relation to service user**	
Spouse	3
Child	1
Parent	1

148

The results reflected four interconnected key themes: 1) Complex System, 2) Knowledge, 3) Stigma and 4) Social cohesion within family and community. Each theme also includes a set of subthemes. These themes and their interconnections are illustrated in Figure 1, and described in further detail below.

Fig 1: Diagram of four key themes and their interconnections

There is some overlap and connection between the four themes (see Fig 1). The themes “Knowledge” and “Stigma” feed into the theme “Social cohesion” and these three themes sit within the wider context of the theme “Complex System”. Furthermore, themes 2 (Knowledge) and 3 (Stigma) interact with each other and there is some overlap and connection between these two themes. For example, the “Stigma” subtheme 3b (Myths about mental illness) does to some extent reflect aspects of the “Knowledge” theme 2. However, the data in theme 3 (stigma) was more specifically related to stigma than the data presented in theme 2.

1. Complex systems

This theme captures the difficulties highlighted by participants in navigating accessing care for their mental health conditions. Participants described seeking help and support for their mental health through a variety of sources and via a number of different routes without clear referral pathways, sometimes simultaneously and sometimes sequentially (subtheme 1a). Participants described barriers at each stage of accessing care and continuing to seek support through the PHC where this study was carried out (subtheme 1b). There was not a single, uniform pathway leading to participants accessing help for their health condition, and several systemic barriers described in accessing consistent healthcare (subtheme 1b). This theme addresses the practical and logistical barriers that have arisen due to the complex system,

rather than barriers in accessing care relating to knowledge and stigma; these are described in themes 2 "Knowledge" and 3 "Stigma", which sit within the context of this theme.

1a. Pathways to care

Participants described frequently seeking access to help and support for mental health conditions through a variety of different sources, including healthcare at the hospital level prior to primary care or alternative sources such as through spiritual care. One caregiver stated:

"I went to different private practitioners in Ramanagara. When it didn't cure kept on seeing different doctors." (SU1)

The experience of seeking care from multiple sources before the primary health centre without one clear linear referral pathway was frequently mentioned, for example:

"*We took her to private hospital and district hospital for treatment initially. We did not know that medicines are available in PHC."* (CG2)

Seeking support from spiritual sources was frequently mentioned either prior to seeking care from health services, or alongside healthcare, as illustrated in the following data extracts:

"We went to temples and did poojas, but had no improvement. Then our neighbours told about taking treatment from Ramanagara District Hospital."* (CG3) (*Pooja is a word used to describe a ritual of respect or worship to a god e.g. visiting a temple, making an offering, chanting prayers).

"I used to do poojas and prayers for her improvement. But there was no change… Later, I took her to District Hospital to get treatment." (CG3)

Some participants continued to seek spiritual support alongside care at the primary health centre*:*

"I did Poojas. There was some relief. Now also pooja gives me some satisfaction." (SU3)

The variety of responses highlighted the heterogeneity in pathways for seeking care for mental health conditions and the lack of awareness of the possibility of attending the primary health centre for mental healthcare referral and follow up, despite it being the usual first point of access for other health issues.

1b. System related barriers to accessing care

Several difficulties with accessing care at the primary health centre level and navigating the system were mentioned by participants including financial barriers, travel difficulties, medicine availability, human resources and long wait times. Many participants highlighted the lack of public transport to the primary health centre and that they must walk or use an autorickshaw, which was described as expensive, adding to the financial burden of seeking healthcare and the difficulty accessing the system:

"We live in village, which is 3 kms far from PHC. It is difficult to reach PHC because there are no public transport facilities." (CG5)

"I do not have money to go to far places. I have to come by auto even to the PHC. I have to bear all expenses." (CG3)

As well as transportation being a barrier to accessing care, the limitations of resources at the primary health centre was highlighted by participants in terms of human resources and medication supplies:

"If there are two or three doctors at PHC and if they are specialized, there will be better services at the primary care centres." (CG1)

"Staffs should be regularly available to offer services to the patients. Especially if doctors are available every day, people will consider and accept care at PHC." (CG1)

"*I have not been getting two medicines. I came twice and the medicine is unavailable. Those are two medicines that makes my daughter normal.*" (CG3)

These barriers described the difficulties service users experienced in accessing the public healthcare system to get support for their mental health. As well as the complex journey to reach the system and the practical barriers to accessing the system participants experienced, they also experienced difficulty once within the system in the way of several limitations creating obstacles to accessing continued support for their mental health. Stigma-related influences constituted another barrier to accessing care, which is discussed in theme 3.

2. Knowledge about mental health

Reflections around how much knowledge service users, their carers and health professionals had around the service user's mental health was a repeated and prominent theme in the data. The concept of "not knowing" in subtheme 2a about one's mental health (e.g. the name of your diagnosis, the name of your treatment, where/how to get further support) was a repeated concept. It is important to highlight that this was not assumed to be negative and participants lack of knowledge was not presented in an overtly negative or positive light (subtheme 2a). It was also noted that the way participants conceptualised their conditions was frequently with relation to physical symptoms and beneficial treatments were often biological (e.g. medication, behavioural activation) as described in subthemes 2b and 2c. Participants also expressed a desire for improving and increasing knowledge for carers and staff (subtheme 2d).

2a. Lack of knowledge

Several participants highlighted that they do not have information about their diagnosis:

"Doctors do not give information about illness. They just give medicines and ask them to take regularly and tells that the problem will be reduced on taking treatment." (CG2)

However, it was not necessarily always the case that participants wanted to request this information from doctors:

"I do not know the name of problem. I did not ask doctor and doctor also did not tell." (CG2)

Half of the participants specifically said they did not know the name of their illness, answering simply *"I don't know."* when asked. Many also said they did not know the cause of their illness, again answering *"I don't know."*

Regarding knowledge about sources of support, when asked if they are treated differently or have a complaint what to do, half of the participants said that they "did not know" what to do.

Although half of participants said they would not know what to do if they had a complaint, some participants were aware of routes to raising concerns or complaints. However only one participant (SU3) stated *"I will complain to District Health Office".* Four others said more generally they would tell staff at the hospital. Two participants said they would not make a complaint, one stating:

"*If I do not like treatment or any services or have any other problems, I will go to other place for treatment. I will not go for any complaints."* (CG5)

2b. Knowledge relating to diagnosis

When asked "What do you call these problems you are getting treatment for?", only one participant was aware of their diagnosis (SU6): *"My problem is called fits."* Another participant said (SU4): "*I have some problem of brain."*

Of the fourteen participants, half did not know their diagnosis, one did know their diagnosis, and the remaining participants described their problems in terms of the symptoms, primarily biological or physical issues:

"*My problems are that [an] angiogram has been done, I have blood pressure, sugar, abdominal pain, leg pain, body pain, sleeplessness, tension, difficulty in breathing."* (SU1)

"I was suffering from problems of headache, sweating, sleeplessness, pass stool for excessive times." (SU2)

"I have chest pain, leg pain and eye pain." (SU5)

Tension was a word frequently used to describe symptoms:

"*She has too much tension."* (CG5)

"Tension is my problem." (SU3)

2c. Knowledge relating to treatment

Participants spoke about the positive benefits of support they have received for their health problems including medication, and alternative therapies. Beneficial treatments included medication, alternative/spiritual support and behavioural activation, rather than reference to psychological therapies.

"She is taking medicines from this PHC. She has also taken help from Ayurveda [system of traditional medicine in India]. *After taking that, there is some improvement."* (CG5)

"We get medicines and there is some improvement." (CG5)

Some participants were actively engaged in maintaining their good health:

"I have received training classes for 1 hour from this PHC on how to lead life, how to sleep well, I have been instructed to do exercise, walking and I do 5 kms walking." (SU3)

When asked whether there are any human rights or advocacy organisations that support people with mental health conditions and their families, all participants said no, or that they did not know of any which is in keeping with the lack of such organisations in this area.

2d. Desire for more knowledge

The importance of specialised mental health training and education to increase knowledge was highlighted by several participants, for both health professionals and for lay people:

"*If we [*carer*] get information on how to take care of mental illness, it will be really helpful."* (CG5)

"Staffs should know about mental illness and how to take care of patients." (CG2)

"PHC staffs are all good and helpful, but I need to tell my problems with a psychiatrist who can only actually help me. We need specialist help and services." (SU3)

3. Stigma

Experiences of stigma and discrimination were described by all the participants. Participants described their observations of negative attitudes from others related to their mental health condition (subtheme 3a) as well as incorrect and stigmatising beliefs around mental health conditions about their treatability or consequences (subtheme 3b). The largest subtheme of this theme was the experiences of negative social and behavioural consequences (discrimination) and being treated differently as a result of their mental health condition (subtheme 3c).

3a. Stigmatising attitudes

Participants described observing negative attitudes from others regarding their mental health condition and the impact this has on them:

"People talk about my illness in a wrong way. How can I control them? I cannot do anything." (SU6)

"Other people think it as a different illness, not like fever or cough." (CG2)

The impact of negative attitudes from others was striking and one participant described the subsequent social isolation that resulted:

"They see us as strange persons. We used to not go out of our home because of this." (CG2)

3b. Myths about mental illness

Some myths about mental health conditions were described by participants, contributing to the stigmatising attitudes described above:

"People think that the illness will not cure." (CG2)

"Sugar can be controlled on taking medicines, but mental illness cannot be controlled." (CG3)

"People think that mental illness cannot be treated." (SU2)

A specific myth about not being able to have children was described:

"*People talk among themselves that I am having some mental problem, tell that I take medicines and I do not get child."* (SU6)

3c. Discrimination

Participants described the subsequent discrimination and ways they are treated by others as a result of their mental health condition, for example being treated differently:

"People behave differently. Some people won't show interest to talk to us while some others fight with us." (SU2)

"People look at persons with mental illness in a different way." (CG3)

A lack of respect was frequently mentioned:

"*My family members do not listen to me and scolds me. Nobody respects me, especially my daughter in law and grandchildren."* (SU5)

"Mostly people in my village treat me with less respect." (SU3)

There was a significant number of mentions of violence or physical mistreatment:

"I have experienced violence from my husband." (SU4)

The social exclusion and rejection from others in light of their mental health condition was frequently mentioned:

"People will remain far from such people who have this illness." (CG5)

Use of stigmatising language was described:

"I had a fight with staffs there one day because they told that I am 'Brain Loose'." (SU9)

A lack of help from others was frequently described: "*No one else is helping."* (CG3)

4. Social cohesion within family and community

The most distressing stigmatising experiences of discrimination presented by participants were most frequently in the context of exclusion from the community and a prominent theme

throughout the interviews was of service users and carers describing their experiences generally in the terms of how it related to their family or caregivers. The involvement of family in supporting with accessing and providing care was frequently described (subtheme 4a) and the subsequent impact on families and carers of the individuals experiences was also frequently described (subtheme 4b). Within this, the initial presentation to services was often described as being due to a breakdown of relationship or conflict within the family or community, highlighting the importance of social cohesion. Rather than seeking care as a result of symptoms, it was more commonly seen that families sought care when disruption to the family cohesion was experienced. Within theme 4 is also data relating to the positive experiences participants described relating to social cohesion (subtheme 4c).

4a. Importance of family

The involvement of family in supporting participants to access care and also take part in activities of daily life was frequently described by participants:

"My family members- wife and son- took me to NIMHANS." (SU8)

"My daughter does not perform any activities of self-care such as eating, toileting, bathing, dressing on her own. I am doing everything for her." (CG3)

The supportive role of family was described for example when participants experience discriminatory behaviour or stigmatising attitudes:

"My wife also does not listen to them [those in the community expressing stigmatising attitudes and behaviours] *and she supports me."* (SU6)

Furthermore, the impact of service user's health conditions on family members was described and participants spoke about their concerns and anxieties for the wellbeing of their family members experiencing difficulties with their mental health:

"I was worried that she became like this. I was taking care of her." (CG2)

"I need my daughter to be well, I wanted to know if she will also become like other children and it would be good if staff can help me to know." (SU2)

4b. Psychosocial impact of mental health condition

Related to the importance of family, participants often described the nature of their problem with relation to the impact it had or was having on their family and community relationships. Often, the reason or initial presentation to services involved a difficulty within the family or community context:

"I am getting tensed when others are arguing with me. It was started when we were building our new house. There were some family issues. Then I got all worries." (SU9)

It was striking to note that anger or a family conflict was frequently cited as the first reason help was sought or the main way the mental health condition presented:

"My mother was okay before two years and anger increased suddenly." (CG1)

"My son fights sometimes and we have worries when he behaves like that thinking why he is behaving like that with us. He does not listen to what we say or give respect to us." (CG4)

"She fights with me and scold in the evening when I come home after work without any reason." (CG3)

Difficulties in the work place context were also a trigger to mental health deterioration as described by one participant:

"I had work place related issues. The colleagues were cheating and taking money. Then, I got lot of pressure and stress and then I felt that my brain has got some problem." (SU4)

4c. Describing positive experiences with healthcare staff

Many participants described good relationships with healthcare staff:

"All staffs and doctor are very cooperative at PHC." (SU3)

"Doctor in this PHC is really helping minded. She takes care well. Other staffs also understand my problems and support me." (SU9)

Contrasting with the negative experiences of discrimination reported by several participants, there were also several comments about not being treated differently and being accepted by communities:

"No one in our village behave in a different way. I did not experience like that. People ask us to do some Poojas and offer prayer in temples to have improvement." (SU7)

"No one has treated him differently till now. No one has caused any trouble to us." (CG4)

Discussion

The aim of this study was to gain an insight into the experiences of stigma and discrimination that service users and their carers attending a primary health centre in rural Karnataka, India, face relating to their mental health care. It is important to understand the stigma-related barriers that individuals in specific contexts face in accessing mental health services to inform the development of locally relevant anti-stigma interventions and improve access to care for people with mental health conditions. In addition, such insights aim to improve quality of life and promote the rights of individuals with mental health conditions. Such studies are particularly needed in LMICs, where research to date is limited and where there are significant treatment gaps for mental health.

This study highlights barriers to accessing care on both a system level and also relating a lack

of knowledge about mental health. This study brings to light the pervasive experiences of discriminatory behaviour due to their mental health condition that individuals in a rural community in India face. Stigmatising attitudes (prejudice) and behaviours (discrimination) were described by participants from within their local communities and family contexts. It was noted that cohesion within the family and community was a priority for participants, and may be considered "what matters most" (2) for the participants in this study. The concept of "what matters most" is considered a helpful framework for understanding the contextual nuance and cultural influences on stigma (2, 19). Social cohesion and connection appears to be a culturally significant aspect of daily life in this study, that is compromised by the threat or experience of stigma and discrimination.

In India, as in most LMICs, many people with severe mental health conditions may be made homeless, or depend on their families financially and for support with personal and health care (20). Families are therefore often closely involved with seeking healthcare and taking on roles that may be filled by health or social care services in other settings (20). India is traditionally viewed as a collectivist society, where values such as social cohesion and community are held as a priority and where interdependence is promoted (21). The value of social cohesion may be even higher in rural settings where cultural values may be more traditional, and studies have shown differences between experiences of discrimination of people with mental health conditions such as schizophrenia in India in rural compared to urban areas (22). The finding of social cohesion and the importance of family being a key theme in this data is therefore in keeping with what is expected from existing literature and knowledge.

Rather than operating as individuals as might be seen in a Western context, participants in this study routinely described their experiences collectively, in relation to their family and community context, whether directly or indirectly. The importance of cohesion and

maintaining relationships within their family or community context was repeatedly highlighted as a priority for participants. An interesting phenomenon noted by the authors was that many participants described positive experiences of their engagement with the primary health centre despite the barriers they also described. One possible explanation is that the importance of maintaining cohesion and good relationships with the professionals who care for them may have influenced participant answers in light of professionals having an ongoing role in their care. The value of social cohesion above a more Western value of autonomy therefore may extend beyond family and society and influence experiences within healthcare settings.

Participants frequently described mental health conditions as presenting initially with a behavioural change causing conflict within the family or some form of dysfunction with relation to participation in society. Rather than a description of depression involving the cognitive symptoms (e.g. low mood, anhedonia, poor motivation, hopelessness, suicidality) more frequently, behavioural and somatic symptoms were described (poor sleep, poor appetite, irritability, conflict) and their impact on their function within the family or social context. Interestingly, no participants described any of their symptoms or illnesses using language relating to thoughts or emotions (e.g. low mood, hopelessness). Furthermore, participants who described helpful aspects of treatment and care referred to biological tools such as medication and a healthy lifestyle.

Previous studies of the experience of stigma and discrimination of people with schizophrenia in India have highlighted that more internalised forms of stigma for example a sense of alienation is a more common experience than negative discrimination (23). In this study, participants described the negative consequences of stigmatising attitudes from others in terms relating to their social isolation and exclusion from community, for example leading to them not leaving the home.

Participants frequently did not know their diagnosis or treatment plan, in contrast with the focus on patient knowledge, autonomy and rights that are often prioritised in a more Western context. The lack of knowledge was observed to be distinct from the experiences of stigma and discrimination that was described by participants, and was therefore separated into a different theme.

There are several implications of this study relating to clinical practice and future research opportunities. This study highlights the experiences of stigma and discrimination that individuals within a rural context in India are experiencing and the need for the development of relevant anti-stigma interventions which take into consideration the importance of social cohesion and also the practical and systemic barriers that are present for individuals to access care. Addressing stigma and discrimination are urgent global issues, which require immediate action (1). Given the size of India and the diversity of its population, it would be beneficial for this research to be replicated with a wider range of participants in a variety of geographical areas across the country. In order to address the potential barrier that participants may face regarding speaking openly about their experiences of healthcare, and given the importance of social cohesion and hierarchical relationship with professionals, it would be beneficial to involve independent researchers separate from healthcare settings in carrying out further studies.

Certain limitations of this study are important to highlight. As interviews were carried out in Kannada and translated to English before being transcribed, the analysis is based on the translator's interpretation of the participants responses, rather than the direct responses themselves. However, the translators were confident in both languages and all translations were subsequently cross checked. The insights gathered from the data remain valuable as despite the limitations, important rich data have been collected relating to the experiences of individuals with mental health conditions in rural India.

The participants of this study shared several similar characteristics; they were all married, all Hindu and from a rural district in India. Therefore the results may not necessarily be able to be generalised. However, qualitative research findings are not intended to be broadly generalisable, but rather intend to explore the experiences and insights of the specific participant group involved in the study. Furthermore, the shared characteristics of the sample strengthens the themes identified in these data as there were many similarities in the participants' context.

Conclusion

The results from this study give a voice to individuals experiencing stigma and discrimination relating to their mental health, and their carers, providing a unique window into the experience of service users and carers from a rural location in India. Service users from LMICs, particularly in rural settings, are a group which are often overlooked, especially in research studies (1). The experiences of stigma and discrimination the service users and carers who participated in this study is vitally important to be recognised. There is a richness to these data that adds greatly to the body of research exploring service-user experience of stigma and discrimination, which is primarily focused on voices from high-income settings.

Acknowledgments

We thank the participants for their time in providing data for this study, and the NIMHANS research team for their support in data collection and translation.

Ethical approval

Ethical approval was obtained from the PNM Research Ethics Subcommittee, King's College, London: Reference Number (No.) RESCMR-17/18-4109 and NIMHANS Institutional Review Board: Reference Number (No.) NIMHANS/IEC(BEH.SC.DIV.)7th MEETING/2017. No further ethical clearance was required, as permission for the use of these data in further studies including secondary analyses was already obtained through the original ethical approval.

References

1. Thornicroft G, Sunkel C, Alikhon Aliev A, Baker S, Brohan E, El Chammay R, et al. The Lancet Commission on ending stigma and discrimination in mental health. Lancet. 2022;400(10361):1438-80.
2. Yang LH, Chen FP, Sia KJ, Lam J, Lam K, Ngo H, et al. "What matters most:" a cultural mechanism moderating structural vulnerability and moral experience of mental illness stigma. Soc Sci Med. 2014;103:84-93.
3. Mak WW, Chan RC, Wong SY, Lau JT, Tang WK, Tang AK, et al. A Cross-Diagnostic Investigation of the Differential Impact of Discrimination on Clinical and Personal Recovery. Psychiatr Serv. 2017;68(2):159-66.
4. Patel V, Saxena S, Frankish H, Boyce N. Sustainable development and global mental health--a Lancet Commission. Lancet. 2016;387(10024):1143-5.
5. Patel V, Saxena S, Lund C, Thornicroft G, Baingana F, Bolton P, et al. The Lancet Commission on global mental health and sustainable development. Lancet. 2018;392(10157):1553-98.
6. Clement S, Schauman O, Graham T, Maggioni F, Evans-Lacko S, Bezborodovs N, et al. What is the impact of mental health-related stigma on help-seeking? A systematic review of quantitative and qualitative studies. Psychol Med. 2015;45(1):11-27.
7. Mehta N, Clement S, Marcus E, Stona AC, Bezborodovs N, Evans-Lacko S, et al. Evidence for effective interventions to reduce mental health-related stigma and discrimination in the medium and long term: systematic review. Br J Psychiatry. 2015;207(5):377-84.

8. Semrau M, Evans-Lacko S, Koschorke M, Ashenafi L, Thornicroft G. Stigma and discrimination related to mental illness in low- and middle-income countries. Epidemiology and psychiatric sciences. 2015;24(5):382-94.

9. Hertog S, Gerland P, Wilmoth J (United Nations Department of Economic and Social Affairs). India overtakes China as the world's most populous country. April 2023. Future of the World Policy Brief No 153.

10. Chokshi M, Patil B, Khanna R, Neogi SB, Sharma J, Paul VK, et al. Health systems in India. J Perinatol. 2016;36(s3):S9-s12.

11. Thara R, Patel V. Role of non-governmental organizations in mental health in India. Indian J Psychiatry. 2010;52(Suppl1).

12. Sagar R, Dandona R, Gururaj G, Dhaliwal RS, Singh A, Ferrari A, et al. The burden of mental disorders across the states of India: the Global Burden of Disease Study 1990–2017. The Lancet Psychiatry. 2020;7(2):148-61.

13. The Mental Healthcare Act, 2017. No. 10 of 2017. Registered no. Dl—(n)04/0007/2003-17. (April 7th, 2017).

14. Mahapatra P, Seshadri S. Mental health in India: evolving strategies, initiatives, and prospects. Lancet Reg Health Southeast Asia. 2024;20:100300.

15. Garg K, Kumar CN, Chandra PS. Number of psychiatrists in India: Baby steps forward, but a long way to go. Indian J Psychiatry. 2019;61(1):104-5.

16. Koschorke M, Oexle N, Ouali U, Cherian AV, Deepika V, Mendon GB, et al. Perspectives of healthcare providers, service users, and family members about mental illness stigma in primary care settings: A multi-site qualitative study of seven countries in Africa, Asia, and Europe. PLoS One. 2021;16(10):e0258729.

17. Clarke V, Braun V. Successful Qualitative Research: A Practical Guide for Beginners. 1st Ed. Sage Publishing Ltd. 2013.

18. Braun V, Clarke V. Using thematic analysis in psychology. Qualitative Research in Psychology. 2006;3(2):77-101.

19. Gurung D, Poudyal A, Wang YL, Neupane M, Bhattarai K, Wahid SS, et al. Stigma against mental health disorders in Nepal conceptualised with a 'what matters most' framework: a scoping review. Epidemiol Psychiatr Sci. 2022;31:e11.

20. Koschorke M, Padmavati R, Kumar S, Cohen A, Weiss HA, Chatterjee S, et al. Experiences of stigma and discrimination faced by family caregivers of people with schizophrenia in India. Soc Sci Med. 2017;178:66-77.

21. Chadda RK, Deb KS. Indian family systems, collectivistic society and psychotherapy. Indian J Psychiatry. 2013;55(Suppl 2):S299-309.

22. Loganathan S, Murthy SR. Experiences of stigma and discrimination endured by people suffering from schizophrenia. Indian J Psychiatry. 2008;50(1):39-46.

23. Koschorke M, Padmavati R, Kumar S, Cohen A, Weiss HA, Chatterjee S, et al. Experiences of stigma and discrimination of people with schizophrenia in India. Soc Sci Med. 2014;123:149-59.

Figure 1

Click here to access/download;Figure;Fig1.tiff

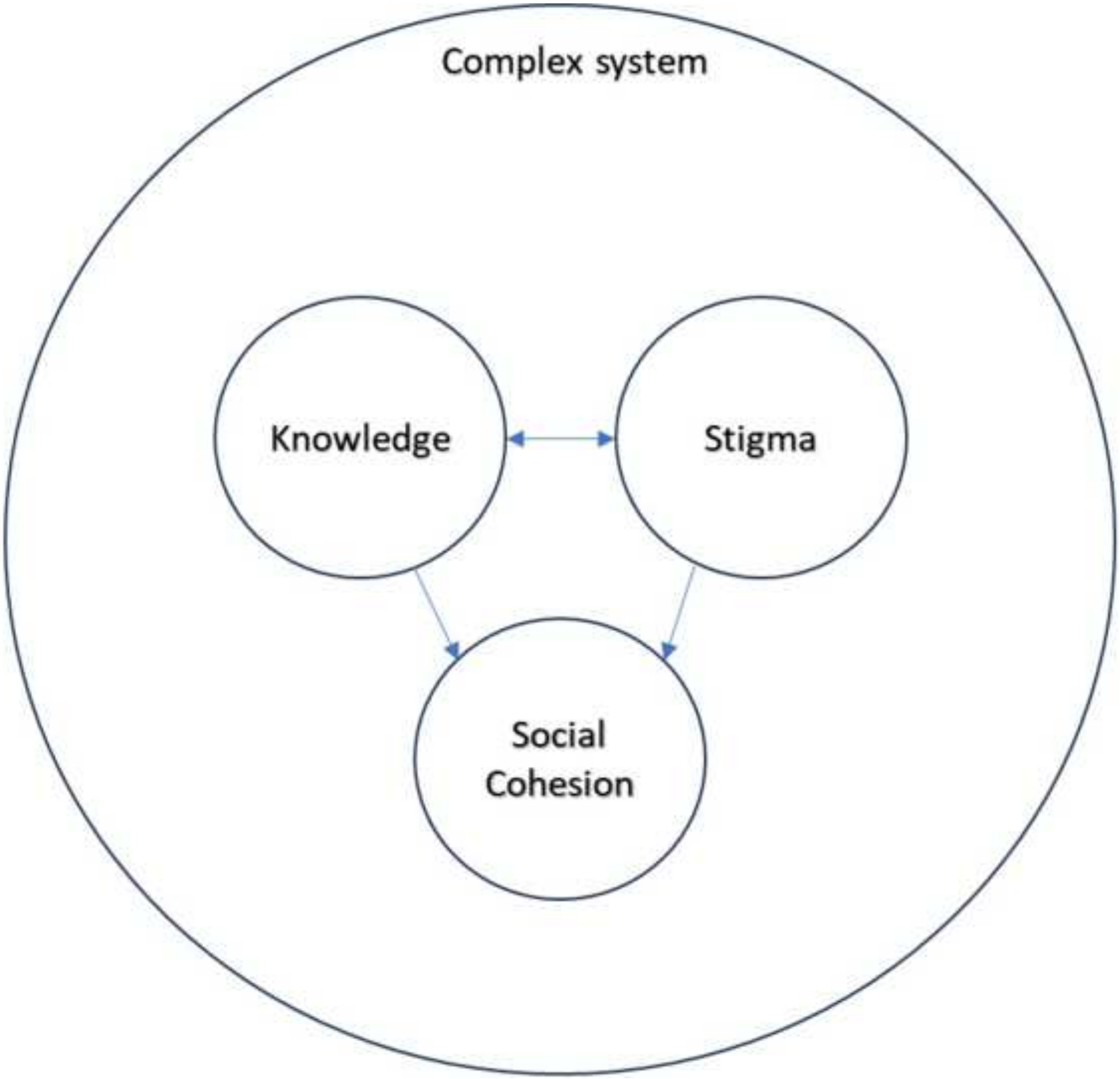

Supplement: Attachment — Submitted filename: PONE-D-24-43039.pdf [file pone.0330949.s003.pdf]
