# Supplementary material for: Mental health related stigma in a primary care setting in Karnataka, rural India: Service user and carer perspectives
Source: PLoS One. 2025 Aug 22;20(8):e0330949. doi: 10.1371/journal.pone.0330949 (PMC12373246; doi:10.1371/journal.pone.0330949)
Supplement: Attachment — Submitted filename: PONE-D-24-43039R1_Response to reviewers_250523.pdf [file pone.0330949.s006.pdf]

Line	Reviewer 1 comments	Response
2	1. The reviewer suggested we edit the title to specify the location as Karnataka, India	Thank you. To reflect this we have changed the title to: “Mental health related stigma in a primary care setting in *Karnataka*, rural India: service user and carer perspectives”
31	2. The reviewer suggests “Including india”	Thank you for this comment. It is not clear to us how this comment should be interpreted. If a specific edit or action is suggested please can this be clarified, and we are happy to consider this.
83	3. In reference to the figure of 200 million people in India in 2017 being estimated to have a mental disorder, the reviewer suggests a more up to date reference	Thank you for this comment. The last National Mental Health Survey in India took place in 2015-2016, therefore the most recent accurate data remains from this time. The survey is being repeated currently, which will generate new up to date data. We hope this explains why this is the most recent accurate reference that was chosen to be included.
107	4. The reviewer suggests extending the abbreviation “PHC” used	Thank you for suggesting expanding the use of the abbreviation PHC. We would like to highlight that we have extended the abbreviation on the previous page on line 77 (line numbers refer to the revised manuscript with track changes), according to the PLOS one manuscript guidelines of defining an abbreviation on first use in the text. We hope that explains the use of this abbreviation here.
113	5. The reviewer made a comment stating: “Methods”	Thank you for this comment. It is not clear to us how this should be interpreted. If a specific edit or action is suggested please can this be clarified, and we are happy to consider this.
144	6. The reviewer suggested removing author contributions from this section: “Analysis was carried out by author SP in collaboration with the three other authors (AC, PCG, GM), to identify themes describing the entire data set. All key themes were developed and agreed on by all four authors over a series of online meetings.”	Thank you for this comment. In the PLOS one submission guidelines for the methods section there is an emphasis on giving sufficient and detailed information about the methods used. We also adhere to ICMJE authorship criteria, for which it is likewise recommended to clarify author contributions. For the purpose of transparency we feel it is helpful for readers to have a sense of which authors were involved with data analysis which was the rationale for including this information. However, if the editor has any preference regarding this then please do let us know as we will adjust accordingly.
158	7. The reviewer recommends revising the results section to includes more depth and synthesis of the context of the study<br>“The result description is shallow and very fragmented. each response has to be well articulated along with the representation of	Thank you for this comment. We aimed to use the results section of our paper as the place to present the analysed data, without synthesis or discussion. These latter elements were reserved for the discussion section. In keeping with how data are typically presented in a research paper, we aimed to keep discussion and comments on the results of data analysis to the discussion section only. Using thematic

	gesture and overall reflection about that issue. it is unstructured to and hard to catch the readers to the study findings and context. I found it mostly a simple copy of response without synthesizing the context of the study along with the its magnificecy. It requires more work and revision."	analysis generates themes as results, which are presented in the results section. We aimed to present the themes in a structured manner, using them as headings to give structure to the section. We do hope this answers the reviewers comments on this section of our paper.
161	8. The reviewer suggests merging tables 1 and 2<br>"Merge table 1 and 2 addition informations communicative frequency too"	Thank you for this comment. We have merged the tables as you suggest, compiling demographic data for the service users and carers into one table, named Table 1, on line 165.
164	9. The reviewer suggests the themes should be described in the methods section..<br>"These theme should be described well in the methods section."	Thank you for this comment. We used a thematic analysis approach to analyse our qualitative data (see ref 20, Braun & Clarke 2006 paper "Using thematic analysis in psychology"). This approach generates themes as the results from data analysis. Therefore the authors felt the most appropriate position for the description of these themes is in the results section, as they are the results of the analysis of the data, rather than part of the methods. We do hope this explains the rationale for themes only being described in the results section.
177	10. The reviewer highlighted the theme "Complex System" and recommends "Discrimination which is one of you variables is missed the system especially in the model image you produced."	Thank you for this comment. In light of your recommendation, we have renamed theme 3: "Stigma and Discrimination" (see line 324) to make the importance of discrimination to this theme more overtly clear. Discrimination was already considered within this theme and its subtheme (especially subtheme 3c: discrimination), however we hope renaming the theme provides even greater clarity. We do hope these changes address your suggestions, thank you.
433	11. The reviewer suggests development of the discussion section<br>"Another major limitation of the paper is this part. it is fragmented and lacks consistency with the themes of the study in coherence. I recommend authors to rewrite theis section just by following the expected revision in the result section and then to revise it carefully."	Thank you for this comment. In order to create more coherence and structure in the discussion section we have added subheadings for "future recommendations" (line 505) and "limitations" (line 520) (see also next comment 12). This clearer structure addresses your comment about the section being fragmented. We have further revised the discussion section by adding references to enhance the coherence of the section, and more clearly linking the themes that emerged from our data with patterns reported in the broader literature.
532	12. The reviewer suggests revision of the conclusion	Thank you for your comment. As also outlined in comment 11 above, we have added a new subheading to the discussion section for "future

	"The conclusion too has to be revised based on the revised result and discussion. The main findings, its implications and future recommendation has to be presented with the frame of the study findings and context, that are lacking in this version."	recommendations" (see line 505) to highlight this element of the discussion.

Line	Reviewer 2 comments	Response
5	1. The reviewer queries the use of & symbol	The & symbol is used to indicate joint last author: Anish Cherian & Petra Gronholm are joint last authors for this manuscript. This is according to the PLOS One title, author, affiliations formatting guidelines. This is now also clarified on the title page. I do hope that clarifies the use of the & symbol, thank you.
17	2. The reviewer points out the duplication of 5 for author affiliations	Thank you very much for pointing out this error, we have corrected the manuscript to reflect the affiliations numbered 5 and 6
63	3. The reviewer highlights use of the word "Thus" in the sentence on line 61 as requiring restructuring	Thank you for this comment. We have restructured this sentence as per your recommendation in order for it to be clearer. We do hope this addresses your comment.
67-71	4. The reviewer questions whether the comments made on lines 65-69 (now lines 67-71) are the authors thoughts	Thank you for this comment and highlighting the omission of the relevant references in this section. In order to provide the relevant evidence base for this section we have added two references.
109	5. The reviewer questions whether the manuscript is suggesting the previous study was not focused and detailed in light of this study intending to carry out a more focused and detailed analysis	Thank you for this comment. The use of the phrase more "focused and detailed" was intended to refer to emphasis on the context in India, rather than suggest that the previous publication, or its analysis, was not focused or detailed. The previous publication had a cross-site perspective, and as such its purpose was not to go into a level of contextual detail or focus on any of the individual sites. To ensure the meaning of this is clear, we have restructured the paragraph to highlight the focus and detail is relating to the context of this setting in India. This now reads:<br><br>Line 106 (line numbers refer to the revised manuscript with track changes): "*The site for this study was in rural India, in Ramanagara district within Karnataka state, at PHCs associated with the District Mental Health Programme*. These data have previously been analysed considering data from all research sites (17). The purpose of this study is to carry out a focused and detailed analysis of the data collected with service-users and carers in the site in India specifically."

113	6. The reviewer highlights further details required in the participants and procedure section:<br>"Kindly provide details on the qualitative methodology and processes? What was the interview scenario? Was probability of relapse considered and were clinical experts and psychologist available to manage if any deterioration occurred? Were these ethical considerations maintained?"	Thank you for suggesting these elements of clarification. Further details have now been added regarding the interview scenario:<br><br>Line 128: " The interviews were conducted in the Kannada language, in private settings where participants could freely express their views".<br><br>We have also added the following elaborations regarding ethical considerations and risk management to the manuscript:<br><br>Line 116: "Recruitment procedures emphasised assessing potential participants' capacity, and the voluntary nature of study participation."<br><br>Line 125: "Interviews were carried out by members of the research team ... experienced in conducting mental health research and trained to recognise and respond to signs of potential distress during data collection."<br><br>These edits reflect how the ethical approval underpinning this research involved a number of risk considerations and mitigation strategies. These included added attention on vulnerable participants providing informed consent, and potential perceived pressure to participate. In view of this recruitment procedures emphasised assessing for capacity, and the voluntary nature of participation. Ethical considerations also extended to the risk of research participation causing psychological distress. The team at NIMHANS are experienced in conducting sensitive mental health research, and researchers received training on how to recognise and respond to signs of potential distress. The participants had all by default already connected with mental health care, as they were recruited through signposting via primary health centre/PHC staff associated with the District Mental Health Programme in the study setting. Any concerns would have been reported to, and followed up as appropriate, PHC staff. No one was actually unwell at the time of participation, as those currently symptomatic were excluded from recruitment.
120	7. The reviewer queries the sample size<br>"10-15 participants per site might have been enough for a multisite study. This study includes 14 participants divided in groups of 9 service users and 5 carers? Does it	Thank you for this question. To clarify, participant recruitment in each site of the original multi-site study was guided by site-level saturation. Thus, the Karnataka sample of 14 participants was assessed as sufficient in view of this principle. For rationale for the sample size, the reference provided with this point (Ref 18, Clarke & Braun, 2013) recommends 10-20

	suffice for data saturation? Repetitions of narrative (extracts) from same participants within the results hints otherwise."	interviews for a "medium project, or a study within a larger project", reflecting this study's sample size. We have now also included a further reference (Ref 19, Guest et al., 2006; , which reports how saturation is generally reached at around 12 interviews.
132	8. "Were the questions same for service users and carers?"	Thank you for bringing to our attention that the supplementary material was missed in the upload. This is now included as mentioned in the manuscript. As seen in the supplementary material, the questions were the same for service users and carers, with adjustments according to the perspective of the respondent (whether service user or caregiver).
146	9. "As the themes were developed in collaboration with the four authors agreement is obvious. Was there any other processes to maintain rigor in the analysis and results?"	Thank you for this comment. We have provided further detail in the data analysis method by expanding the methods section to highlight in greater detail the processes used to maintain rigor in the analysis. The positionality of the four authors involved in the data analysis is also important to highlight. Two of the authors involved in analysis work within the context of the study, whereas two authors were external to the data collection process and work in an entirely different context. This range of perspectives reduced bias in the analysis whilst maintaining important knowledge of the context. This section now reads:<br><br>Line 138: "The transcribed data were inputted to NVivo (R14.23.2) to facilitate data analysis. The data were examined through thematic analysis (20). An inductive method of thematic analysis was used. This process involved an initial familiarisation and immersion in the data, followed by generating a set of initial codes, which were then analysed and organised. Themes were identified from the codes through an iterative process and a series of online meetings between authors. The themes were refined to final key themes and a visual representation of the thematic map created as part of this process (20). Analysis was carried out by author SP in collaboration with three other authors (AC, PCG, GM), to identify the key themes describing the entire data set. Two authors (AC, GM) work within the study context, and two authors (SP, PG) work externally to the setting. This range of perspectives reduced bias in the analysis whilst maintaining important knowledge of the context. All key themes were developed and agreed on by all four authors over a series of online meetings."
433	10. The discussion section seems more of opinion from the fourth paragraph with minimum compare and contrasts. This might require	Thank you for highlighting further references are needed within this section. Our comments reflect our interpretation of the findings as related to the field, references have been added throughout to provide

	reviewing. Also the ante-penultimate paragraph seems to be more of conclusion and recommendation.	relevant evidence, which our findings are contrasted with and related to.<br><br>We have revised the section to clearly show the content of the last three paragraphs of the discussion and added subheadings: *Future recommendations* (line 505) and *Limitations* (line 520). This was also recommended by Reviewer 1: comments 11 and 12.
538	11. do we require citations in conclusion? might require some rethinking. Also user perspectives are considered in many qualitative studies in India, Nepal and other south east asian countries, kindly review literature and compare and contrast in the discussion section.	Thank you for this comment. We are happy to remove the reference in the conclusion section if this is not according to PLOS One guidelines.<br><br>We have added additional references to the discussion section including perspectives in other South Asian and Southeast Asian contexts. We have drawn a comparison to other contexts in Asia (see line 451).
