# Supplementary material for: Mental health related stigma in a primary care setting in Karnataka, rural India: Service user and carer perspectives
Source: PLoS One. 2025 Aug 22;20(8):e0330949. doi: 10.1371/journal.pone.0330949 (PMC12373246; doi:10.1371/journal.pone.0330949)
Supplement: Attachment — Submitted filename: PONE-D-24-43039R1_R2_Response to reviewers_250804.pdf [file pone.0330949.s007.pdf]

Reviewer #1

Comments for the manuscript titled "Mental health related stigma in a primary care setting in Karnataka, rural India: service user and carer perspectives". The authors have provided a "Response to previous round of review and revisions, which is a positive sign of engagement with feedback. However, a thorough review against PLOS ONE's criteria reveals several areas requiring improvement. While the study addresses a valid research question, clarity, methodological rigor in reporting, and comprehensive engagement with existing literature need to be strengthened.

AUTHORS RESPONSE: We thank the reviewer for their careful attention to our manuscript, and have provided responses to their specific queries below.

1. Literature Review: The current version of the manuscript should be carefully re-evaluated to ensure the literature review comprehensively places the claims in context and fairly treats prior work.

AUTHORS RESPONSE: We have reviewed the literature cited in the introduction section, and have added further references (especially reviews and recent studies, where available), to provide a more comprehensive overview of literature providing the context for this research.

We have also reviewed the presentation of this background section, and confirm the past literature is fairly treated, without any prior conclusions being misrepresented or key sources or diverging views being omitted.

Updates to citations added through the reference manager do not show up in track changes, but we confirm the added references are the following:

- 2. Thornicroft G, Mehta N, Clement S, Evans-Lacko S, Doherty M, Rose D, et al. Evidence for effective interventions to reduce mental-health-related stigma and discrimination. Lancet. 2016;387(10023):1123-32.
- 3. Semrau M, Evans-Lacko S, Koschorke M, Ashenafi L, Thornicroft G. Stigma and discrimination related to mental illness in low- and middle-income countries. Epidemiology and psychiatric sciences. 2015;24(5):382-94.
- 5. Yang LH, Kleinman A, Link BG, Phelan JC, Lee S, Good B. Culture and stigma: adding moral experience to stigma theory. Soc Sci Med. 2007;64(7):1524-35.
- 7. Zhang Z, Sun K, Jatchavala C, Koh J, Chia Y, Bose J, et al. Overview of Stigma against Psychiatric Illnesses and Advancements of Anti-Stigma Activities in Six Asian Societies. International Journal of Environmental Research and Public Health. 2020; 17(1).
- 11. Henderson C, Evans-Lacko S, Thornicroft G. Mental illness stigma, help seeking, and public health programs. Am J Public Health. 2013;103(5):777-80.
- 12. Vaishnav M, Javed A, Gupta S, Kumar V, Vaishnav P, Kumar A, et al. Stigma towards mental illness in Asian nations and low-and-middle-income countries, and comparison with high-income countries: A literature review and practice implications. Indian J Psychiatry. 2023;65(10):995-1011.

Methods:

2. The manuscript should clearly detail the specific qualitative methodology used (e.g., thematic analysis, grounded theory) and justify its selection.

AUTHORS RESPONSE: The manuscript specifies that thematic analysis was used for the analysis (p.6 line 137). A further sentence has now been added as rationale for this approach (p.6 line 138):

"… allowing themes to emerge directly from the data without being influenced by pre-existing theories, enabling a rich, data-driven understanding of participants' experiences."

3. More extensive details on the interview guide, how interviews were conducted (e.g., duration, setting, language, who conducted them), and how data saturation was determined would enhance reproducibility.

AUTHORS RESPONSE: Regarding the interview guide, the manuscript already provides examples of the topics within the interview guide (p.6 line 128) (*"an interview guide addressing topics related to experiences of stigma within healthcare and community settings for service users and carers"*), and the full interview guide is provided in Appendix 1 (as signposted on p.6 line 131).

Regarding interview conduct, the manuscript already provides details on their duration (p.6 line 128: *"Interviews lasted 60-90 minutes"*), setting (p.6 line 126: *"in private settings where participants could freely express their views"*), language (p.6 line 126: *"The interviews were conducted in the Kannada language"*, and p.6 line 131: *"The interviews were translated and then transcribed in English by the same researcher. A further researcher, fluent in both English and Kannada crosschecked the transcript with audio recordings and reviewed the translation independently."*), as well as who conducted them (p.6 line 123: *"members of the research team from National Institute for Mental Health and Neurosciences (NIMHANS), experienced in conducting mental health research and trained to recognise and respond to signs of potential distress during data collection"*).

Regarding data saturation, the manuscript (p.5 line 117) already outlines, with references, that *"a sample of around 10-15 participants per research site was assessed as sufficient to reach data saturation"*. We have now added the following sentence to further describe how saturation was determined (p.5 line 119):

"Saturation was considered achieved when the interviews were deemed to not provide new information or insights, indicating that additional data collection is unlikely to further the understanding of the research question.

4. The data analysis process needs to be explained with greater rigor. This includes details on coding procedures, theme development, and how trustworthiness (e.g., credibility, transferability, dependability, confirmability) was ensured.

AUTHORS RESPONSE: The word count restrictions for the original submission prevented us from elaborating on the details of the analysis process. However, we have now added the further details to expand the description in the 'data analysis' paragraph of the methods section, for added clarity on the coding process and theme development (p.6 line 139) (added text indicated with underlined font):

"This process involved an initial familiarisation and immersion in the data through repeated reading of the transcripts, followed by generating a set of initial codes representing emerging broader patterns and descriptors of the raw data. These ~~which~~ were then analysed and organised, through considering clustering between similar codes and connections between these broader groupings. Themes were identified from the codes, reflecting latent constructs characterising these emerging code clusters. These emerging themes were refined through an

iterative process of re-reading transcripts, revisiting the codes and themes and their connections, reviewing and refining the code and theme names for more accurate representation of the experiences and constructs they captured. The lead author (SJP) led this process, and the emerging insights and thematic structure was discussed in a series of online meetings between authors."

We have also added the following paragraph to illustrate how this analytical process aligns with Nowell et al.'s guidance on practical procedures for meeting Lincoln and Guba's trustworthiness criteria in studies using thematic analysis, p.7 line 157:

"Following practical guidance for achieving trustworthiness criteria in thematic analysis conduct (Nowell et al. https://journals.sagepub.com/doi/10.1177/1609406917733847), the analytical procedures described above also attest to how trustworthiness was established in this research. For example, the analytical process involved prolonged engagement with the data, retained field notes and transcripts, documenting the evolving coding process through notes shared amongst the research team, researcher triangulation during coding, audit trail of code generation, diagramming to make sense of code generation, vetting themes and subthemes among the research team, team consensus of themes, and providing a detailed description of the process of coding and analysis. "

5. Ethics Statement: Ensure it clearly states how informed consent was obtained.

AUTHORS RESPONSE: The ethical approval statement details the specific approvals granted for this work, and including details on procedures for obtaining consent is not relevant within this section. However, details on obtaining informed consent are included in the 'Participants and procedure' section of the methods (p.5 line 115): *"Participation in the study was initiated only after written, informed consent had been obtained."*

6. Data Availability: The manuscript needs to explicitly state where the data can be found, or if there are restrictions (e.g., due to participant privacy in qualitative data), these must be clearly specified and justified. For qualitative studies, this often involves depositing anonymized transcripts or a detailed data dictionary and codebook in a recognized repository.

AUTHORS RESPONSE: We have corresponded with the PLOS ONE Editorial Team regarding the data availability for this manuscript, and emails dated 17 June, 2025 (email trail available on request) confirmed that the data availability statement – as formulated below and submitted with the manuscript - is approved for this study:

"Public deposition of the data underlying our findings is not possible due to ethical reasons, as consent was not sought from participants to having their data shared (even if anonymised) through methods like public repositories or as a supplement to the manuscript, and as such sharing data in this way would breach compliance with the protocols ap-proved by the relevant research ethics boards that have approved this research (King's College London PNM Research Ethics Subcommittee). Rather, participants have consent to their data being available for others through requests to the project team, i.e. the manuscript authors, or alternatively via contacting the King's College London Research Ethics Office (rec@kcl.ac.uk). Anonymised data will be made to other researchers via request, and no reasonable request will be denied."

As further context, PLOS asks that authors provide a statement which explains where and how a manuscript's minimal data set can be found. The minimal data set is defined as the data set used to reach the conclusions drawn in the manuscript with related metadata and methods, and any additional data required to replicate the reported study findings in their entirety. This may include: a.) The values behind the means, standard deviations and other measures

reported; b.) The values used to build graphs; c.) The points extracted from images for analysis (https://journals.plos.org/plosone/s/data-availability#loc-minimal-data-set-definition). As our study used qualitative methods, no values etc. can be provided as a minimal data set. We have, however, provided at least one narrative participant quote for each subtheme of the results, illustrating the data that supports these. We consider these numerous participant quotes the minimal data set, and confirm that this is available within the submitted manuscript.

Results

7. Support for Conclusions: Ensure the results directly answer the research questions posed in the introduction.

AUTHORS RESPONSE: To more clearly link the results to the research question posed in the introduction, we have reiterated the research question in the results section, p.9 line 184 (added text indicated with underlined font):

"The aim of this study was to explore the experiences of stigma and discrimination of service users and carers attending a primary health centre in rural Karnataka, India for mental health conditions. The results reflected four interconnected key themes relating to this research question: 1) Complex systems…"

Discussion

8. Interpretation and Comparison: The discussion section should interpret the findings in light of previous research and their implications. The "Response to Reviewers" mentions that the discussion was revised to reflect the authors' interpretation of findings as related to the field. This is a good improvement, but the reviewer should critically assess if these comparisons are robust and if the discussion avoids being overly opinionated without sufficient comparative analysis.

AUTHORS RESPONSE: We have reviewed the discussion section, ensuring the comparisons made between the current findings and past literature are fair and do not make unsupported interpretive claims. The added methodological details added in response to Question 4 further supports the robustness of the findings reported in relation to the previous research.

We have also included additional references that provide a stronger basis for comparing our findings with existing literature. These additions aim to enhance the robustness of our interpretations and ensure that our discussion is grounded in relevant empirical evidence, thereby avoiding overly subjective or opinion-based assertions.

Updates to citations added through the reference manager do not show up in track changes, but we confirm the added references are the following:

- 12. Vaishnav M, Javed A, Gupta S, Kumar V, Vaishnav P, Kumar A, et al. Stigma towards mental illness in Asian nations and low-and-middle-income countries, and comparison with high-income countries: A literature review and practice implications. Indian J Psychiatry. 2023;65(10):995-1011.
- 7. Zhang Z, Sun K, Jatchavala C, Koh J, Chia Y, Bose J, et al. Overview of Stigma against Psychiatric Illnesses and Advancements of Anti-Stigma Activities in Six Asian Societies. International Journal of Environmental Research and Public Health. 2020; 17(1).

Reviewer #2

The manuscript reads better after addressal of the comments from the reviewers. However, there are some major comment that needs to be highlighted:

AUTHORS RESPONSE: We thank the reviewer for their careful attention to our manuscript, and have provided responses to their specific queries below.

1. It seems like the current study methodology is reviewing of the transcript available from previous multinational study (DOI: 10.1371/journal.pone.0258729). Therefore, the methods section may require rewriting as it is not a new study but using data and information from prior study. Consider secondary information review type of methodology highlighting the information availability through previous study.

AUTHORS RESPONSE: Correct, the methods section starts by stating that this study considers data collected as a part of the earlier INDIGO Primary study. As such, this study is not posited as a new study. We have also not positioned this work as secondary data analysis, as this would not be an accurate representation of our study. Namely, we are not reanalysing data originally collected for a different purpose to answer a new research question. Rather, both the original broader INDIGO Primary study and the current study considers the same broad research question (understanding mental health stigma and discrimination in primary care settings). However, the current study enables a richer, more in-depth exploration of this question through a) a focus on the Karnataka site in India specifically, compared to the earlier multi-site study; b) analysis using inductive thematic analysis, compared to the previous framework analysis of these data alongside data from other sites; and c) a focus on a specific subset of the participant sample (service-users and carers), compared to the broader full sample considered in previous analysis (primary care providers, service users, and their families as well as other stakeholders).

We have now revised the initial 'study design' paragraph of the methods section to be clearer about how the work reported in this manuscript relates to the original broader study (p.5 line 99) (added text indicated with underlined font):

"This study used data collected as part of the Indigo PRIMARY study (22), a multisite qualitative study of experiences of stigma relating to mental health conditions in primary care settings across Asia, Africa and Europe. Methodological details for this broader study have been reported previously (22). The site considered for this study was in rural India, in Ramanagara district within Karnataka state, at PHCs associated with the District Mental Health Programme. These data have previously been analysed using framework analysis techniques, considering data ~~from~~ across all research sites (22). The purpose of this study is to carry out a ~~focused~~ richer, more in-depth exploration of these data, ~~and detailed~~ though an inductive analysis of the data collected with service-users and carers in the site in India specifically."

2. Intercoder reliability or similar objective approaches would strengthen the rigor of the study rather than only speaking of agreeing upon on meetings (subjective approach).

AUTHORS RESPONSE: The manuscript reports on how the analysis process was not subjectively conducted by a single author, but rather reflected a collaboration between multiple co-authors (p.7 line 151):

"Analysis was carried out by author SJP in collaboration with three other authors (AC, PCG, GM), to identify the key themes describing the entire data set."

Additionally, in response to Reviewer 1's Question 4, we have added further details to demonstrate the rigour of the thematic analysis process, alongside details on how trustworthiness was established in the analysis process. These added details demonstrate the transparent process underlying the analysis process, and how the analysis process was not dependent on one author's subjective position but rather involved elements of coding and feedback from other co-authors.

We now also include the following statement in the limitation section, p.24 line 545:

"We also acknowledge the limitation that for feasibility reasons, the analysis did not include independent coding by multiple authors or procedures to establish intercoder reliability. However, the lead author collaborated with other authors during the analysis process, ensuring coding was not biased by a subjective perspective."

3. The recommendations fall into conclusion section, not discussion section. Also, conclusion does not take any references.

AUTHORS RESPONSE: We have removed the reference from the conclusion as per the reviewer's request.

We structured our sections according to the PLOS One submission guidelines and also in response to our previous reviewers' comments. We believe that the discussion section is clearer to navigate when the recommendations are included as a standalone section, rather than embedded within the conclusions, and with the conclusion retained as a brief overall summary of the study results and their impact. However, we are happy to follow editorial guidance regarding formatting and whether a longer conclusion section with the recommendation section included is preferred.
